# Targeted delivery of cytotoxic proteins to prostate cancer via conjugation to small molecule urea-based PSMA inhibitors

**DOI:** 10.1038/s41598-021-94534-5

**Published:** 2021-07-21

**Authors:** O. C. Rogers, D. M. Rosen, L. Antony, H. M. Harper, D. Das, X. Yang, I. Minn, R. C. Mease, M. G. Pomper, S. R. Denmeade

**Affiliations:** 1 The Department of Pharmacology and Molecular Sciences, The Johns Hopkins University School of Medicine, Viragh Building, 201 N. Broadway, Baltimore, MD 21287 USA; 2 The Department of Oncology, The Johns Hopkins University School of Medicine, Viragh Building, 201 N. Broadway, Baltimore, MD 21287 USA; 3 The Department of Radiology, The Johns Hopkins University School of Medicine, Viragh Building, 201 N. Broadway, Baltimore, MD 21287 USA

**Keywords:** Cancer, Drug discovery, Oncology

## Abstract

Prostate cancer cells are characterized by a remarkably low proliferative rate and the production of high levels of prostate-specific proteases. Protein-based toxins are attractive candidates for prostate cancer therapy because they kill cells via proliferation-independent mechanisms. However, the non-specific cytotoxicity of these potent cytotoxins must be redirected to avoid toxicity to normal tissues. Prostate-Specific Membrane Antigen (PSMA) is membrane-bound carboxypeptidase that is highly expressed by prostate cancer cells. Potent dipeptide PSMA inhibitors have been developed that can selectively deliver and concentrate imaging agents within prostate cancer cells based on continuous PSMA internalization and endosomal cycling. On this basis, we conjugated a PSMA inhibitor to the apoptosis-inducing human protease Granzyme B and the potent Pseudomonas exotoxin protein toxin fragment, PE35. We assessed selective PSMA binding and entrance into tumor cell to induce cell death. We demonstrated these agents selectively bound to PSMA and became internalized. PSMA-targeted PE35 toxin was selectively toxic to PSMA producing cells in vitro. Intratumoral and intravenous administration of this toxin produced marked tumor killing of PSMA-producing xenografts with minimal host toxicity. These studies demonstrate that urea-based PSMA inhibitors represent a simpler, less expensive alternative to antibodies as a means to deliver cytotoxic proteins to prostate cancer cells.

## Introduction

Prostate cancer is eventually fatal once it has escaped the prostate gland, resulting in the deaths of > 33,000 American men annually^1^. Androgen ablation has been the standard therapy for metastatic disease since its discovery in the 1940’s and represents the first “targeted cancer therapy”^2^. However, while androgen ablation provides substantial palliative benefit, all men eventually develop “castration resistant prostate cancer” (CRPC)^3,4^. A hallmark of CRPC compared to other tumor types is the remarkably low proliferative rate of < 5.0% per day for prostate cancer cells within lymph node or bone metastases^5,6^. This rate is significantly lower than the rate for many normal tissues including the GI tract, the skin and the bone marrow. Unfortunately, because of this unique property, CRPCs tend to be highly resistant to traditional chemotherapeutics that target mitosis such as DNA alkylating agents and antimetabolites. These agents do little to target tumors while generating a large array of off-target cytotoxicity. Remarkably, androgen ablation selectively kills the non-proliferating epithelial cells that make up the majority of cells within the normal prostate and sites of metastatic disease. Therefore, newer agents are needed that, like androgen ablation, can target the > 95% of prostate cancer cells within a given metastatic site that are not immediately proliferating.

Protein toxins represent a class of therapeutic agents that are non-specific, highly potent and proliferation-independent cytotoxins that are primarily derived from bacterial sources^7^. Since the majority of cells in the body are proliferatively quiescent in a G0 state, to avoid severe adverse effects to the host these protein toxins must be redesigned to selectively eliminate slowly proliferating CRPC cells while remaining inactive against normal cells. This goal can be achieved in prostate cancer because these cancers arise from the prostate gland, a sexually differentiated tissue that is non-essential to life, which also produces a number of therapeutic prostate-tissue specific protein targets. These targets include the serine protease Prostate-Specific Antigen [PSA] and the membrane bound carboxypeptidase Prostate-Specific Membrane Antigen (PSMA).

PSMA is a membrane protein that is functionally a glutamate carboxypeptidase II with N-acetylated -linked acidic dipeptidase (NAALDase) activity that is abundantly expressed by prostate cancer cells^8–10^. Its expression is further increased in higher-grade cancers, metastatic disease, and hormone-refractory prostate carcinoma^10,11^. Previous studies have documented that PSMA undergoes internalization via clathrin-coated pits mediated by a novel MXXXL motif in the cytoplasmic tail^12,13^. Thus, PSMA-targeted agents become concentrated intracellularly within the endosomal compartment of PCa cells. These features make PSMA an attractive target for imaging and therapeutic application. Potent urea-based PSMA inhibitors with high picomolar to low nanomolar Ki values have been previously described^14,15^ with ‘next-generation’ inhibitors demonstrating impressive PSMA binding and pharmacokinetic profiles^16^. Radiolabeled version of these PSMA inhibitors have been extensively characterized using SPECT and PET-based imaging modalities in in men with prostate cancer^17–19^. Such inhibitors have also been used successfully in a therapeutic context when conjugated to radionuclides^20,21^. Preclinical studies documented that, similar to labeled anti-PSMA antibodies, upon PSMA binding these compounds are rapidly internalized into endosomes^22^. Thus, they have proved highly selective and sensitive agents for imaging prostate cancer at earlier stage and smaller volumes then possible with conventional imaging with CT, MRI or bone scan.

These PSMA inhibitors, therefore, could target cytotoxic protein payloads for uptake by prostate cancer cells. In order to evaluate the potential for selective delivery we conjugated a urea-based PSMA-inhibitor to two cytotoxic proteins, human Granzyme B (GZMB) and a cysteine-containing fragment of the pseudomonas exotoxin A gene (PE35). Granzyme B is a serine protease secreted by activated cytotoxic T lymphocytes that able to cleave a myriad of cytoplasmic pro-apoptotic and anti-survival substrates thus inducing cell death^23^ GZMB’s function is dependent on perforin-facilitated endocytosis and translocation from the target cell endosome to the cytoplasm^23,24^. Extracellular GZMB can also remodel the extracellular matrix and cause apoptosis by cleaving important matrix proteins^25,26^. PE35 is an engineered version of pseudomonas exotoxin A that is unable to bind to cells due to deletion of the cell targeting domain Ia structure but maintains the ability to disrupt target cell translation via ADP-ribosylation of the crucial diphthamide residue on Elongation Factor 2^27,28^, thus inhibiting general protein translation. In this study we have conjugated a urea-based PSMA-inhibitor to GZMB and PE35 and evaluated the ability of these conjugates to bind and inhibit PSMA, to internalize selectively into PSMA-expressing cells, and to kill prostate cancer cells in vitro and in vivo in a PSMA-specific manner.

## Results

### Production and biochemical characterization of MU2-conjugated protein toxins

**Figure 1:**
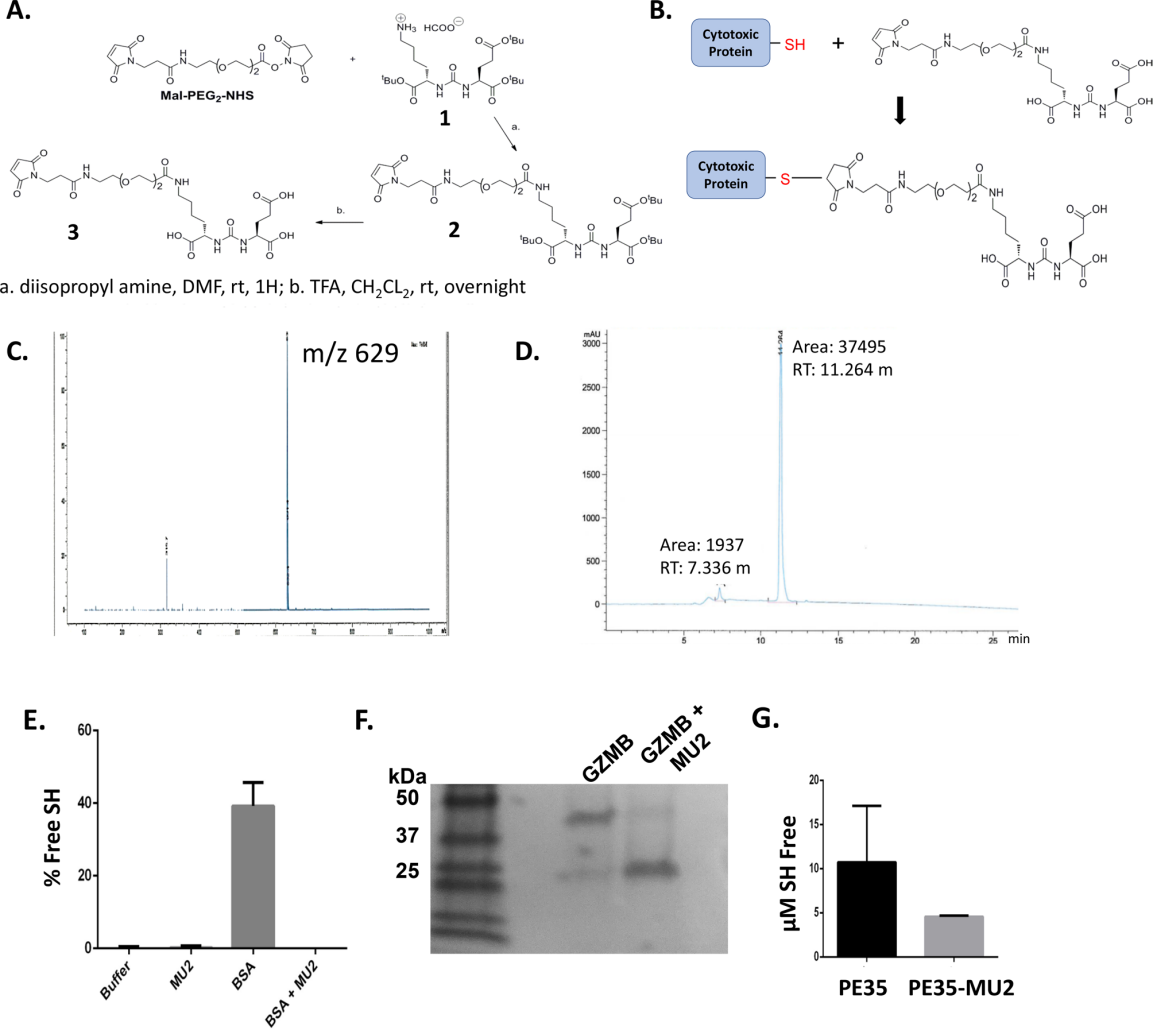
Synthesis and production of protein-urea drug conjugates. (**A**) Chemical synthesis scheme taken to make a thiol-reactive maleimide-linked urea separated by two PEG units (MU2). (**B**) Diagram depicting the protein-MU2 conjugation reaction. (**C**) Mass spectrometry and (**D**) HPLC plot of MU2 product. (**E**) Ellman’s reagent assay of BSA +/− MU2 under non-reducing conditions. (**F**) Non-reducing SDS PAGE gel of C-terminal reactive GZMB +/− MU2 following dialysis. (**G**) ABD-F fluorescent assay of PE35 +/− MU2.

Using the urea-based PSMA-inhibitor scaffold, we synthesized a readily conjugatable, thiol-reactive PSMA-inhibitor that can be attached to free cysteines via a short polyethylene glycol (PEG) linker. This was done by reacting the free amine of the side chain of the PSMA-inhibitor with a maleimide-containing linker via a reactive N-Hydroxysuccinimidyl ester (Fig. 1A). These two reactive groups were linked by 2 PEG subunits and yielded a urea-based PSMA-inhibitor with a maleimide functional group (MU2). Cysteine reactive mutants of GZMB and PE were generated in house via mutagenesis or acquired from the Pastan laboratory respectfully. MU2 conjugation can be readily accomplished at pH 7–8 in aqueous buffered solutions to preserve protein stability and function, (Fig. 1B). Mass spectrometry and HPLC of the compound showed the expected molecular weight of MU2 with a single major peak with a m/z value of 629 that was relatively pure (Fig. 1C, D).

To assess the ease and feasibility of MU2-protein coupling we first coupled MU2 in the presence of the thiol-free reducing agent TCEP to Bovine Serum Albumin (BSA) that contains a readily reactive, external cysteine. Ellman’s assay analysis showed that approximately 40% of cysteine 34 in BSA is reactive at pH 7.4 in aqueous solution under non-reducing conditions. Following overnight incubation of MU2 and protein, we detected no free BSA thiol in solution via Ellman’s Assay (Fig. 1E). To confirm successful coupling of MU2 with GZ-C248, we ran both unconjugated and coupled protein through ion exchange columns, dialyzed overnight, and performed non-reducing SDS PAGE. Once stained, gel analysis showed that dialyzed unreacted GZ-C248 exists in both dimeric and monomeric states with the majority of the material linked via disulfides. GZMB incubated with MU2, conversely, existed in primarily the monomeric state at the expected molecular weight of 28 kDa (Fig. 1F). Lastly, we used a thiol-reactive, fluorescent ABD-F assay to confirm coupling between MU2 and PE35. This assay showed that under reducing conditions, uncoupled PE35 protein [14 μM] had a free sulfhydryl content of 10 μM while the MU2-coupled PE35 had a free sulfhydryl content of 4 μM (Fig. 1G) although this result was not statistically significant due to high variability seen in the uncoupled protein. These results suggest that the completeness of the MU2 coupling is highly dependent on the reactive cysteine available and its microenvironment. Specifically, for PE35, roughly half of the protein appears conjugated compared to untreated free PE35.

**Figure 2:**
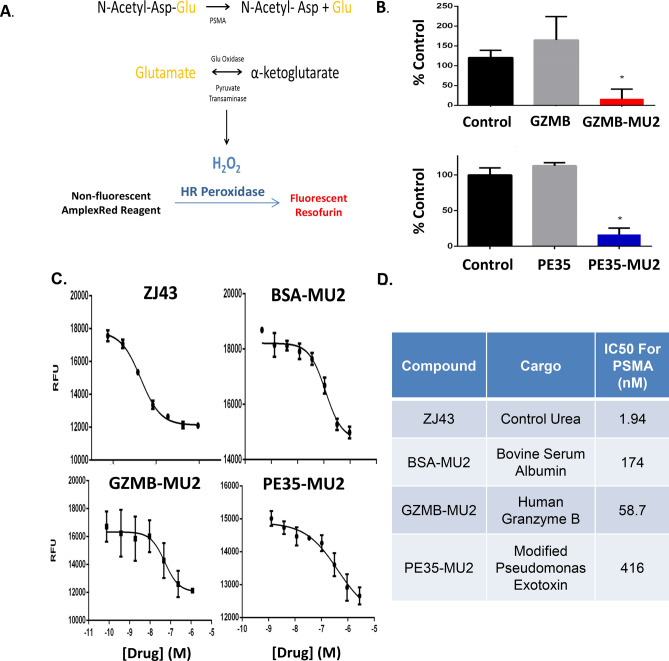
Protein-urea conjugates bind and inhibit PSMA. (**A**) Scheme of the enzyme-coupled PSMA enzymatic assay utilized to detect urea-conjugate binding. (**B**) Inhibition of PSMA by coupled or naked cytotoxic proteins represented as a percentage of the control reaction. (**C**) Dose response curves of ZJ43, BSA-MU2, GZMB-MU2, and PE35-MU2. (**D**) IC50 values for PSMA inhibition obtained for each compound.

To assess the ability of Protein-MU2 conjugates to bind to PSMA, an enzyme coupled assay to assess whether the conjugates could inhibit cleavage of the PSMA substrate NAAG to NAA and free glutamate (Fig. 2A). When compared to buffer-treated controls, both unconjugated GZ-C248 and PE35 proteins had no effect on PSMA enzymatic activity in this assay. However, GZMB-MU2 and PE35-MU2 both inhibited PSMA hydrolysis at 1 μM (Fig. 2B). For all three protein-MU2 conjugates (GZMB, PE35, BSA), we observe a dose dependent inhibition of PSMA (Fig. 2C) with IC50 values in the nanomolar range for all three protein conjugates. However, conjugation to proteins increased the IC50 30–200 fold compared to an unconjugated control urea-based PSMA inhibitor, ZJ43, which has an IC50 of 1.9 nM (Fig. 2D). It is likely that the incomplete conjugation of PE35 led to this higher IC50 value suggesting that the actual IC50 of PE35-MU2 is lower if comprehensive coupling was achieved.

**Figure 3:**
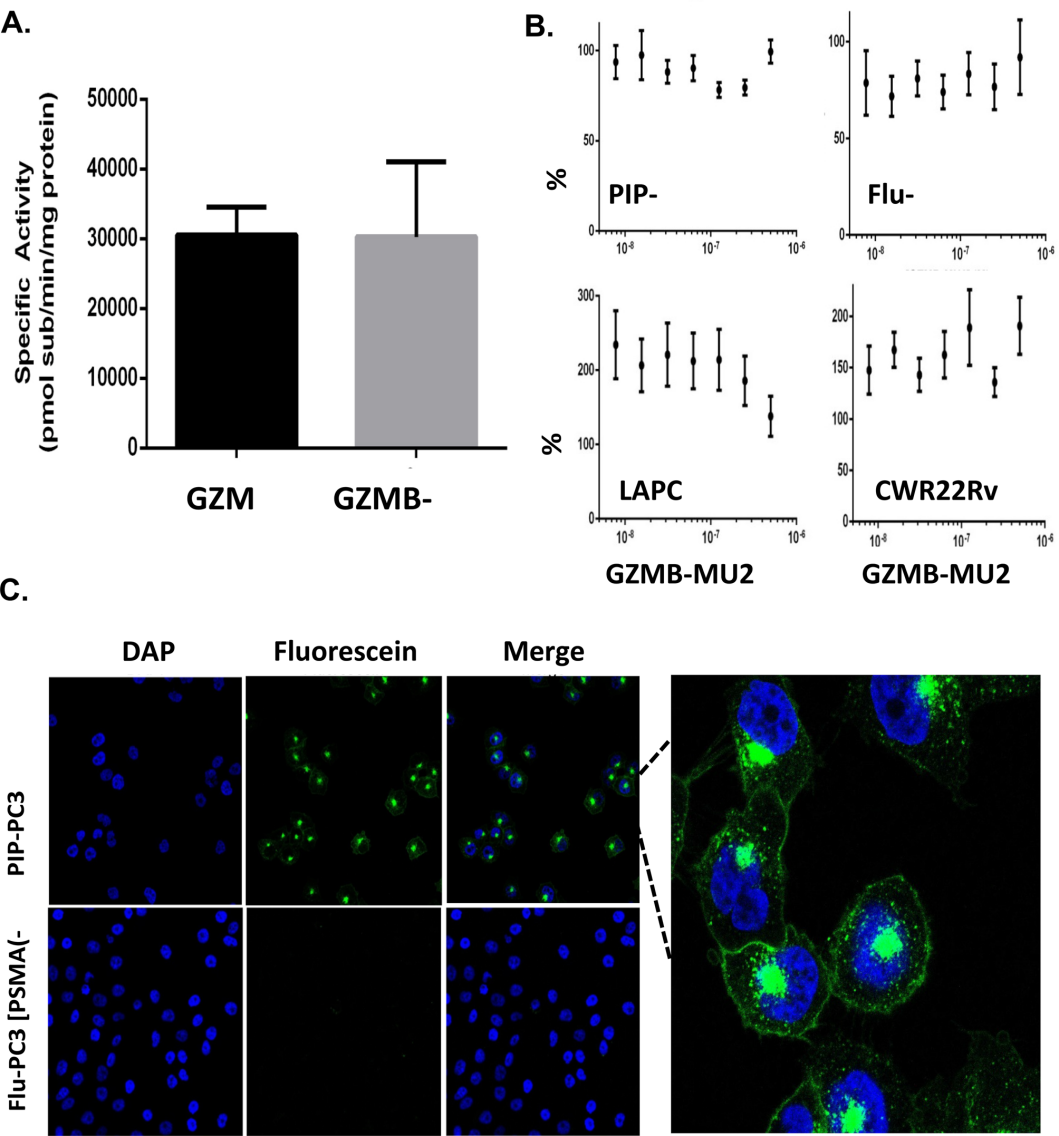
GZMB-MU2 internalizes into PIP cells but does not induce cell death. (**A**) Enzymatic activity of GZMB or GZMB-MU2 using a GZMB-specific fluorescent substrate. (**B**) Cytotoxicity of purified GZMB-MU2 on PIP-PC3, Flu-PC3, LAPC4, or CWR22 Rv1. (**C**) Confocal microscopy of PIP or Flu-PC3 cells treated with Flor-GZMB-MU2 for 1 h at 20 × magnification (left) or 60 × (right).

PSMA-binding assays confirmed that the MU2 urea portion of the conjugate was still functional following conjugation. To assess whether protein functionality was affected by this modification, a GZMB-specific proteolysis assay was performed to compare the functionality of unconjugated GZ-C248 to GZMB-MU2. Both proteins retained proteolytic activity with no difference observed in specific activity per mg protein between these two proteins indicating that MU2 could be readily conjugated without altering protein function (Fig. 3A).

### Cytotoxicity and uptake of GZMB-MU2

The cytotoxicity of GZMB-MU2 was assessed using the previously characterized human prostate cancer cell line PC-3 engineered to express PSMA (PIP) or vector control (Flu) and the PSMA-expressing human prostate cancer cell lines LNCaP and CWR22Rv1. When tested at doses up to 300 nM, GZMB-MU2 had no specific effect on proliferation, cell morphology, or viability in any of prostate cancer cell lines (Fig. 3B). To assess whether the lack of toxicity was due to lack of internalization of GZMB-MU2, a fluorescein-tagged GZMB-MU2 was generated. PIP and Flu cells were exposed to this agent 100 nM for 2 h then co-stained with DAPI to contextualize any observed uptake. After a 1 h incubation, using confocal microscopy we observed positive staining on the PIP-PC3 cells in the fluorescein channel but not on the Flu-PC3 cells. The staining on the PIP-PC3 cells appears most punctate and associated with the DAPI stain with some association to the membrane (Fig. 3C). At 60 × magnification we observe compartmentalization of the signal in the PIP cells as both a large focus adjacent to the nuclear space of the cell as well as smaller foci distributed throughout the cytoplasm of the cell (Fig. 3C). Co-treatment of endosome disrupting HIV TAT peptides^29^ and the small molecule chloroquine^30^ did not lead to increased cell killing of either cell line although it must be noted that robust toxicity was seen when both lines were treated with the latter alone making any effects of GZMB-MU2 difficult to assess (data not shown). It is possible that while these agents may have released a fraction the protein conjugate into the cytosol of PIP-PC3 cells, the levels of which were simply not high enough to activate apoptotic pathways. Serine protease inhibitor (SERPIN) expression by these cells may have also inactivated the catalytic activity of the GZMB component of the conjugate.

### Cytotoxicity and uptake of PE35-MU2

**Figure 4:**
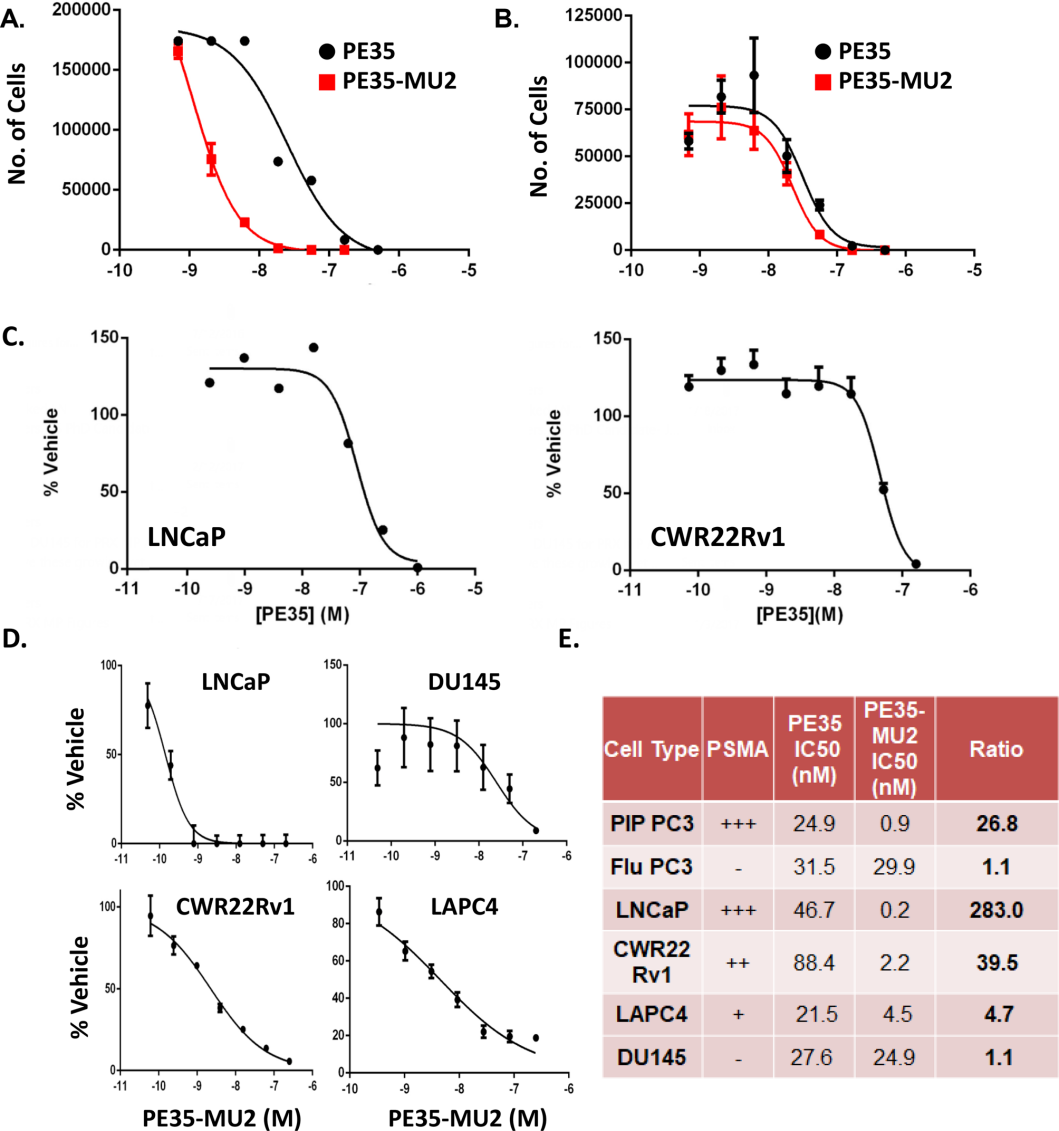
PE35-MU2 is selectively toxic to PSMA producing cells. (**A**) Viability of PIP-PC3 or (**B**) Flu-PC3 cells treated with PE35 (black) or PE35-MU2 (red). (**C**) Dose response curve LNCaP and CWR22 Rv1 to free and PE35 proteins (**D**) Dose response curve LNCaP, DU145, CWR22 Rv1 or LAPC4 to conjugated PE35 proteins. (**E**) IC50 values obtained on various cell lines with unconjugated or conjugated PE35.

PE35 is a potent, non-specific toxin that was equally active against both PIP and Flu-PC3 cells with IC50 of ~ 30 nM against both lines. Conjugation of PE35 to MU2 significantly improved the potency by almost 30-fold against PIP-PC3 with an IC50 of 0.9 nM. However, the toxicity of PE35-MU2 was unchanged compared to PE35 against Flu-PC3 (Fig. 4A, B). Unconjugated PE35 was cytotoxic to LNCaP and CWR22Rv1 cells with IC50 of 46.7 nM and 88.4 nM respectively, (Fig. 4C). PE35-MU2 was also 5–280 fold more potent compared to PE35 against naturally PSMA-producing cell lines LNCaP, CWR22 Rv1 and LAPC4 (Fig. 4D). The fold enhancement correlated with PSMA expression with a 280-fold enhancement against LNCaP cells, which produce the highest levels of PSMA (Fig. 4E). In contrast, no enhancement was observed for PE35-MU2 against PSMA-negative human DU145 prostate cancer cells (Fig. 4D).

**Figure 5:**
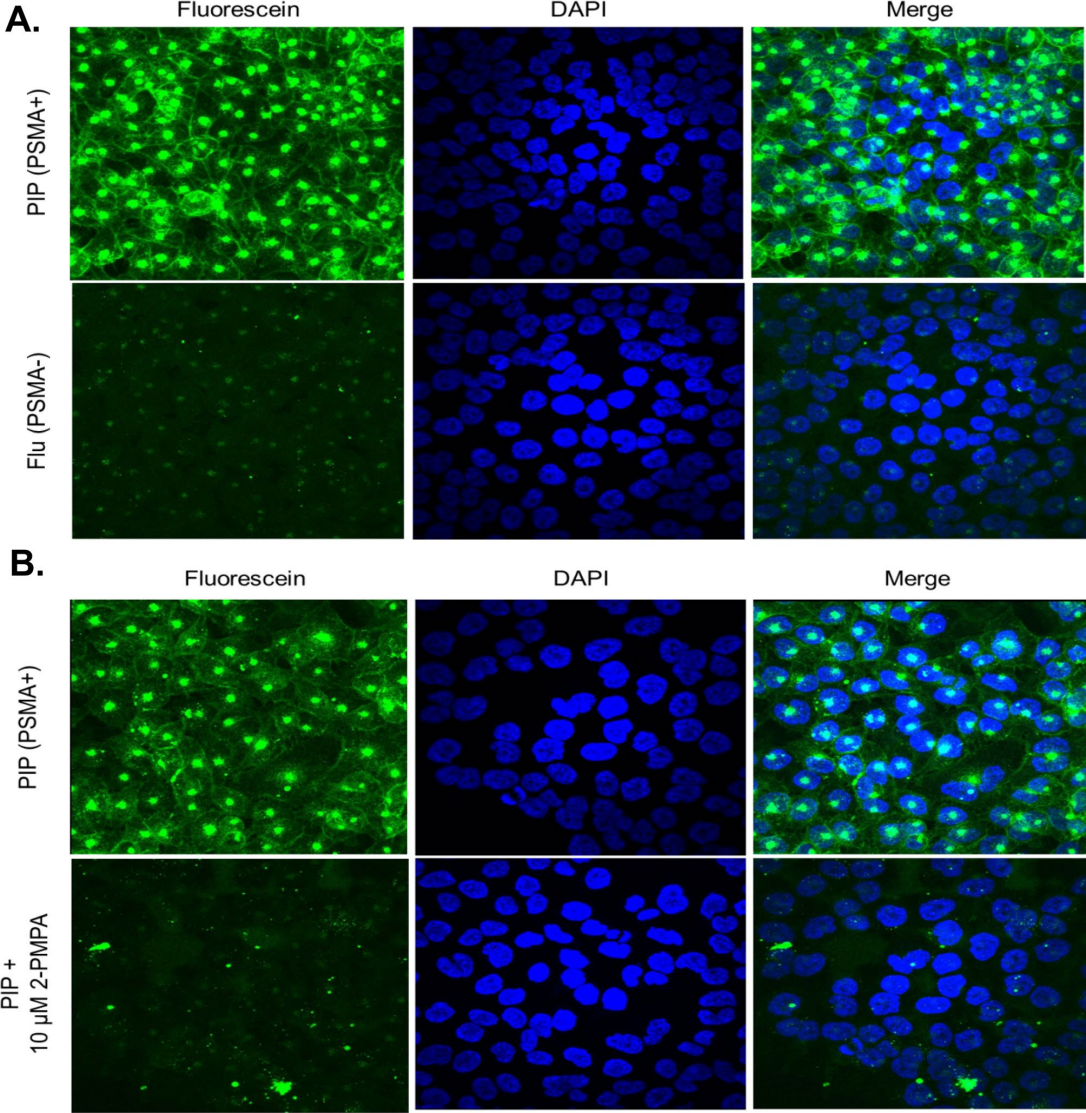
PE35-MU2 is selectively internalized by PSMA expressing cells. (**A**) Confocal microscopy images of PIP-PC3 or Flu-PC3 treated with Flor-PE35-MU2 for 1 h. (**B**) PIP-PC3 cells treated with Flor-PE35-MU2 plus or minus 10 μM 2PMPA.

We generated Flor-PE35-MU2 to assess uptake of PE35-MU2 using confocal microscopy. After a 2-h incubation at 100 nM, we observed low-level uptake in Flu-PC3 compared to vibrant labeling of the PIP-PC3 cells. Similar to the effect seen with GZMB-MU2, we observed a punctate staining pattern that appears to stain both the plasma membrane and endosomes (Fig. 5A). To determine whether this uptake was PSMA specific and not merely a result of a differential uptake between PIP and Flu-PC3 cells, we treated PIP cells with either the Flor-PE35-MU2 probe alone or the probe with 10 μM 2-PMPA, a potent competitive inhibitor of PSMA that occupies the same binding pocket as MU2. Co-incubation with high concentration of PMPA completely abrogates fluorescein labeling on these cells (Fig. 5B).

### In vivo activity of PE35-MU2

**Figure 6:**
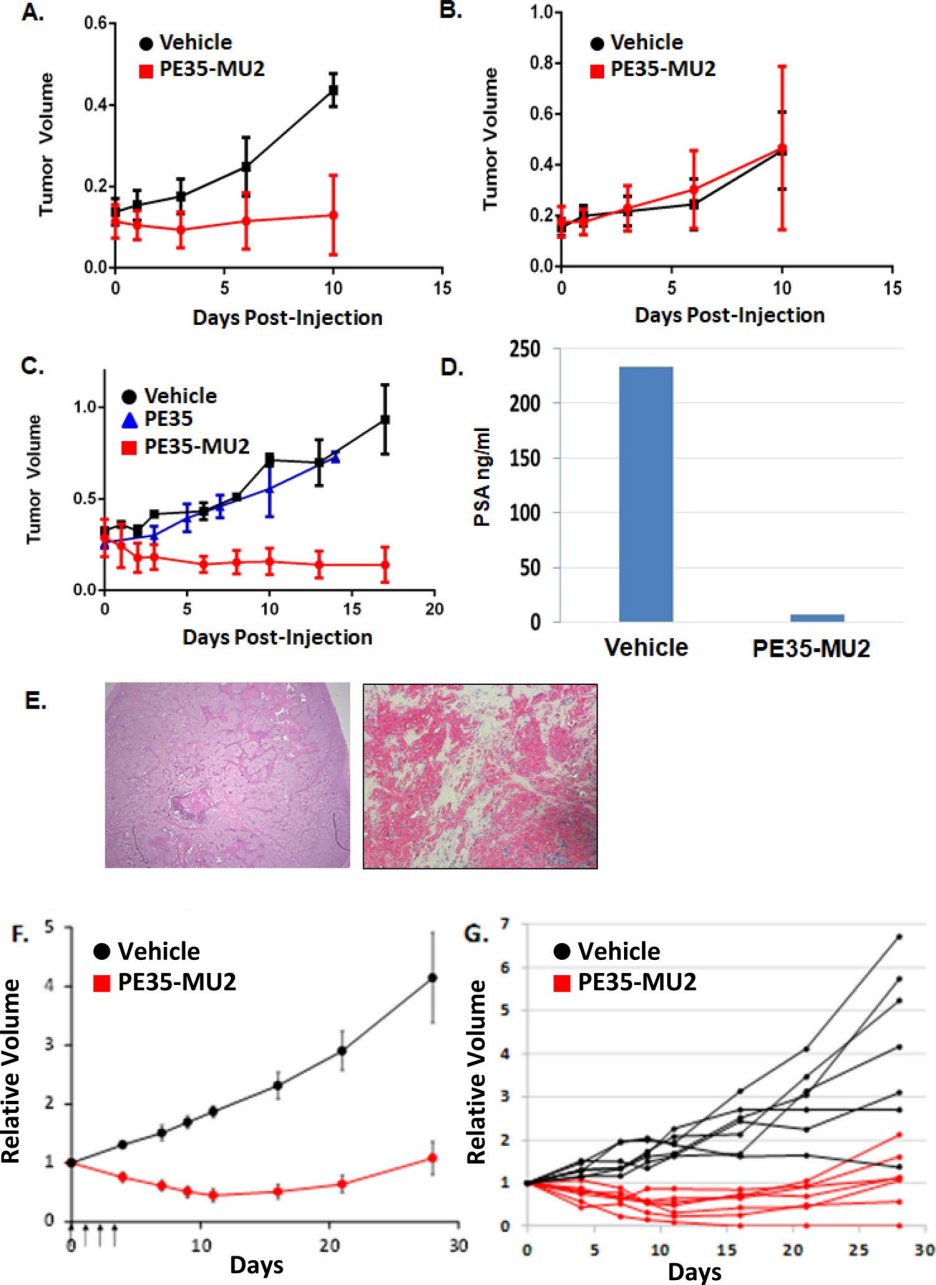
PE35-MU2 regresses PSMA expressing xenografts when injected intratumorally or intravenously. (**A**) Growth of PIP-PC3 (n = 6) and (**B**) Flu-PC3 (n = 6) following two intratumoral injections of vehicle or 20 μg PE35-MU2 (* = *p* < 0.05). (**C**) LNCaP xenograft growth following two injections of either vehicle, 20 μg uncoupled PE35 or 20 μg PE35-MU2 (n = 7 each) (* = *p* < 0.05). (**D**) PSA levels determined via ELISA of LNCaP-bearing nude mice after 3 weeks of treatment. (**E**) H & E staining of an untreated LNCAaP tumor (left) versus a PE35-MU2-injected LNCaP tumor after 3 weeks treatment at (right) (10 ×). (**F**) Growth of LNCaP xenograft following four daily intravenous injections of 50 μg (2 mg/kg) PE35-MU2 (n = 7) versus vehicle (n = 7) (*p* =  < 0.05 at all time points by students t-test). (**G**) Growth curves for individual treated animals.

Previous studies with targeted pseudomonas exotoxin-immunoconjugates revealed a relatively short half-life^31,32^. Therefore, as an initial in vivo assessment of toxicity and efficacy, mice bearing prostate cancer xenografts were treated intratumorally with PE35 or PE35-MU2 to ensure tumor targeting of the agents. Two injections of 0.8 mg/kg of PE35-MU2 significantly inhibited the growth of PSMA[+] PIP-PC3 cells vs control (Fig. 6A) but had no effect on growth of PSMA[−] Flu-PC3 cells (Fig. 6B). Intratumoral injections of unmodified PE35 or vehicle control had no effect on the growth of LNCaP cells, whereas PE35-MU2 treatment produced > 50% average reduction in tumor size at 2 weeks post-injection (Fig. 6C). PE35-MU2 treatment produced a > 90% reduction in serum PSA levels in LNCaP-bearing animals (Fig. 6D). H & E staining of treated xenografts revealed extensive necrosis throughout the treated tissue (Fig. 6E). Overall, intratumoral treatment with 0.8 mg/kg of PE35-MU2 was well tolerated with no animal deaths and less than 5% body weight loss compared to baseline (Supplemental Fig. 1).

Having established that PE35-MU2 could produce significant antitumor effect when directly targeted to tumors we next generated sufficient PE35-MU2 to explore whether sufficient therapeutic index was achieved through PSMA targeting to achieve an antitumor effect following intravenous dosing. An initial toxicology study demonstrated that a single dose of 2 mg/kg was relatively well-tolerated (< 15% body weight loss and no animal death) whereas doses of 3 mg/kg and 6 mg/kg produced ~ 15% and 30% body weight loss respectively at 1 week post dosing. In an initial experiment, LNCaP-bearing mice (n = 6) received 2 mg/kg intravenous dosing for two consecutive days. This dosing regimen produced an average reduction in tumor volume of 15% at 2 weeks post treatment (Supplemental Fig. 2). In a subsequent study, dosing duration was increased to 2 mg/kg for four consecutive days. This regimen produced ~ 50% average reduction in tumor volume with all treated mice experiencing some degree of antitumor effect at 2 weeks post-therapy (Fig. 6F, G). With this 4-day dosing regimen, no treatment-related deaths occurred. PE35-MU2 treated animals had a maximum average decline of 15% body weight compared to 11% in the control treated animals. Weight loss peaked at 2 weeks and by 2 weeks post-treatment animals had returned to baseline weight.

## Discussion

In this work, we demonstrate that a small molecule urea-base PSMA-inhibitor has the capacity to selectively, rapidly, and efficiently deliver cytotoxic protein payloads to PSMA [+] cells. The conjugation method involved formation of a thioester bond between cysteine-containing variants of the protein toxins GZMB and PE35 and a thiol reactive, maleimide-linked PSMA-inhibitor. We selected these two proteins for these proofs of concept studies due to their diverse mechanisms of action, the requirement for intracellular delivery for cytotoxicity, and the previous studies showing successful delivery when fused to other targeting moieties such as antibodies. Using fluorescent and gel-based assays, we confirmed that coupling of our synthesized urea-based PSMA-inhibitor could be achieved using simple reaction conditions. For both proteins, we determined that coupling occurred at high yield and did not affect protein function. Thus, the ease of the chemistry suggests that the formation of conjugates with other cysteine-containing protein is trivial and that a similar strategy could be employed for a diverse profile of therapeutic proteins.

Using an enzyme-coupled PSMA activity assay, we observed inhibition of PSMA by three different purified protein-urea conjugates. This assay traced the conversion of NAAG, a physiologically relevant and specific PSMA dipeptide substrate, to NAA and free glutamate and correlated PSMA inhibition with urea binding. None of the unconjugated proteins tested had any effect on PSMA activity across a range of concentrations. Although coupled to these large proteins BSA, GZMB and PE35, the PSMA inhibitor maintained the ability to inhibit PSMA activity IC50 values ranging from ~ 50 to 500 nM. However, this degree of inhibition is 25–250 fold higher than that observed for the uncoupled PSMA inhibitor ZJ43 use as a control. This is likely due to steric issues such that the urea molecule will have varying degree of accessibility to the PSMA catalytic site based on the structure of the coupled protein.

Previous studies have demonstrated that PSMA is constitutively internalized via clathrin-coated pits and internalizes to the perinuclear recycling endosomal compartment (REC)^33,34^. While we demonstrated that GZMB-MU2 was capable of inhibiting PSMA and was rapidly and specifically internalized by PSMA-expressing PIP-PC3 cells, the conjugated protein had no effect on PSMA[+] cell viability at concentrations below 100 nM. The punctate and non-diffuse staining pattern on the confocal images strongly suggests that GZMB-MU2 is being sequestered in the endosome of the cell and in the perinuclear recycling endosomal compartment. Intracellular localization of GZMB is crucial for its function because the primary substrates of the protease are located in the cytoplasm. Thus, while the GZMB-MU2 was efficiently targeted to the endosomal compartment, the lack of endosomal escape of this protein into the cytoplasm appears to be the limiting factor preventing cytotoxicity of this agent.

Because the GZMB-MU2 molecule was unable to escape the endosomal compartmentalization following PSMA-mediated internalization, we opted to explore an alternative approach. PE35 is a circularly permutated form of the Pseudomonas exotoxin A (PE) that contains amino acids 280–364 and 381–613 of PE^27^. PE35 contains a single cysteine residue at position 287 that can be used to conjugate the toxin to targeting moieties such as monoclonal antibodies. Following internalization of PE35 into endosomes, the protein is then translocated into the cytosol where it catalytically ADP-riboslylates elongation factor II, inhibits global protein translation, thus leading to apoptotic cell death. Both PE35 and a similarly constructed PE mutant known as PE38 have been conjugated to a variety of targeting antibodies/antibody fragments/cytokines^27,28,31,32,35^. Recently, moxetumomab pasudotox consisting of PE38 conjugated to an anti-CD22 antibody received FDA approval for treatment of hairy cell leukemia^36–39^. The production of this immunotoxin required significant reengineering of the anti-CD22 antibody to stabilize the molecule and improve yields during the purification process^31,34^. Activation of this conjugate requires both proteolysis and disulfide bond reduction to release the antibody prior to translocation of the activated toxin into the cytosol.

PE35-MU2 can inhibit PSMA activity, albeit at a concentration that is tenfold higher than that observed for GZMB-MU2. The PE35-MU2 Conjugate selectively and potently killed PSMA-expressing prostate cancer lines at low nanomolar concentrations and was ~ 30-fold more toxic to PSMA[+] vs. PSMA[−] prostate cancer cells. Using a fluorescent PE35-MU2 probe, we observed specific uptake in PSMA[+] PIP-PC3 cells but not in PSMA[−] Flu PC3 cells whose only biological difference is production of the PSMA protein. To further confirm that this uptake was PSMA-driven and not an artifact of the cell lines, we showed disruption of PE35-MU2 internalization using 2-PMPA, a potent PSMA inhibitor.

Previous studies with antibody-based immunotoxins demonstrated that these proteins have a relatively short half-life of 20–120 min although many factors including molecular weight, charge, shape, and volume, can greatly alter biodistribution especially in the context of renal clearance. That said, due to the lower molecular weight of PE35, these short half-lives are likely driven by filtration by the kidney and proteolytic degradation^40,41^. Dosing levels of these toxins in the range of 0.3–1 mg/kg are generally well tolerated with higher doses producing significant weight loss and death within 2 weeks of dosing^31,32^. Based on the potential short half-life, the intratumoral (IT) route of administration was initially employed to determine whether PE35-MU2 could produce an antitumor effect in vivo. A well-tolerated dose of 0.8 mg/kg × 2 resulted in significant antitumor effect against PSMA[+] PIP-PC3 cells and LNCaP with pathology showing almost complete elimination of tumor cells within 1–2 weeks following injection. In contrast, injection of PE35-MU2 into PSMA[−] Flu-PC3 cells and injection of unconjugated PE35 into LNCaP cells produces no measurable antitumor effect. These results indicate the requirement for PSMA production to mediate toxicity. Intravenous administration of PE35-MU2 was well tolerated after single injection of 1 mg/kg but some weight loss was observed at 3 mg/kg and severe weight loss and death at 6 mg/kg. Prior studies with PE-based toxins showed antitumor effect only using dosing regimens of 3–5 days/week^31,32^. Two consecutive doses of PE35-MU2 at 2 mg/kg produced a minor antitumor effect whereas 4 consecutive doses produced a significant antitumor effect. This dosing regimen did not result in animal deaths, however, it did produce ~ 15% loss of body weight over a 2 week period. Thus, these results demonstrate that the small molecule urea-based PSMA inhibitor can target a systemically administered protein toxin to a prostate cancer site. However, similar to other PE-based targeting approaches, the therapeutic index is narrow with non-specific toxicity likely arising through capillary leak syndrome induced by the PE35 toxin. This was the major toxicity observed in Phase I trials of a PE38-based immunotoxins^36^. This work demonstrates a proof of concept that PSMA-inhibitors can serve as delivery vectors for large cytotoxic cargos. Further studies will be performed to comprehensively characterize the in vivo potential of PE35-MU2 by examining the effects of these bioconjugates on various tissue types and mechanisms of toxicity with focus being on the effects on the liver, spleen, and kidney. The conjugate’s pharmacokinetic profile, as well as optimizing the PSMA inhibitor conjugation chemistry will also be explored.

In summary, this small molecule PSMA-targeted delivery approach has many advantages compared to strategies using monoclonal antibodies, growth factors, or single chain variable fragments. In contrast to complicated strategy to engineer stable and functional antibody-based immunotoxins, the MU2 conjugated toxins are readily generated in high yield using simple chemistry and gentle reaction conditions. Coupling the MU2 moiety does not affect protein function. Subsequent proteolysis or disulfide bond reduction is not required for toxin activation. The MU2-protein immunotoxin is smaller in size than antibody based toxins and may penetrate tumors more readily. The conjugate is also more stable to degradation by non-specific proteases. While this study provides preliminary data to support this targeting approach, further optimization is required. PE35 is a potent bacterial toxin that is immunogenic and has off-target side effects. Reducing off-target toxicity and immunogenicity could be achieved by reengineering the bacterial toxin to be less immunogenic^42^ or by redirecting a human protein toxin to sites of prostate cancer. The thiol-ester linkage formed by the cysteine-maleimide linkage can also be disrupted via a sulfhydryl replacement reaction with free albumin in vivo^43^. Recently developed, more-stable linker technology may help to remedy this problem^44^. The PSMA inhibitor can be further engineered to introduce linkers of varying hydrophobicity, size, and chemical modality to improve steric issues reduce PSMA binding. In addition to high-level expression in prostate cancer cells, PSMA is also known to be expressed by the neovasculature of most solid tumors^45,46^. Therefore, this PSMA-inhibitor approach has the potential to be a broadly applicable method to deliver novel pharmaceutic agents to treat human cancers.

## Materials and methods

### Materials

#### Synthesis of maleimide-linked PSMA-binding ureas for conjugation

The methods for synthesis of MU2 are described in Supplemental information and as previously described^47^. All reagents were purchased from Sigma Aldrich unless stated otherwise.

#### Cloning of GZMB gene and cysteine 248 mutagenesis

The GZMB expression construct was designed as described by Gehrmann et al.^48^. The enterokinase activated GZMB mutant was generated by modifying the native GZMB via the Q5 Site Directed Mutagenesis Kit [E0554S] and the primer set F: CGACAAAATCATCGGGGGACATGAGG R: TCGTCGTCTGCATCTGCCCTGGGCAG. First, we removed the native two amino acids on the N-terminus of GZMB and replaced them with an enterokinase substrate, DDDDK. This was shown to improve expression yields and minimize toxicity on HEK-293T cells. After cloning the expression-competent mutant, we inserted a C-terminal reactive cysteine based on a previous study in which active GZMB immunotoxins were produced with the targeting moiety on the C-terminus using the primers F: CAGTGTGGTGGAATTCATGCAACCAATCCTGCTTC R: GATATCTGCAGAATTCTTAGTAGCGTTTCATGGTTTT. The resulting gene product was then cloned into the mammalian expression vector pcDNA3.1 using an In-Fusion HD cloning kit (Clonetech 638909) according to manufacturer’s instructions. The correct product was harvested and sequenced using the Sanger method via the Johns Hopkins Sequencing and Synthesis core facility.

#### Production of GZ-C248 and PE35

Based on previously described method, we expressed GZ-C248 in HEK-293T cells that were transiently transfected with 25 μg of plasmid mixed with 1.1 mL of OPTI-MEM media and 74 uL of FuGene HD lipofectamine reagent (Promega E2311)^48^. PE35 has been modified to include a reactive cysteine near the N-terminus of the protein^27^. The PE35 plasmid was generously donated by Dr. Ira Pastan. This plasmid was transformed into BL21 DE3 E Coli. To generate a semi-pure PE35 preparation, periplasmic extracts from transformed E Coli were generated by osmotic shock. The resulting extract was eluted from a Sepharose Fast Flow anion exchange resin (GE Healthcare 17–0510-10) using 4 fractions of 0.5 mL PBS pH = 6.5 containing 1 M NaCl and then dialyzed in preparation for conjugation.

#### Protein-MU2 conjugations and chromatography

In order to maximize the efficiency of protein-urea coupling, proteins were gently reduced prior to coupling. The non-sulfur containing reducing agent TCEP was incubated at 0.5 mM with the protein of interest for 1 h to disrupt inter-protein disulfide dimer formation prior to conjugation to MU2. A solution of MU2 (compound 3, Fig. 1A) in water was prepared and pH adjusted to 6–7 prior to mixing to final concentration of 1 mM in the reduced protein solution. This mixture was then incubated at room temperature for an hour and then at 4° overnight. Prior to conjugation, GZ-C248 was activated using EK to release the fully functional enzyme. Purified EK from porcine intestine was diluted in a solution of 10 mM TrisHCl and 10 mM CaCl_2_ and diluted to a solution of 5 EU/mL and digested for 30 min at 37°. Conjugation to MU2 produced GZMB-MU2 which was purified with removal of excess MU2 and EK using cation exchange chromatography using a negatively-charged SO3-resin (Fractogel EMD SO3-Millipore 116882). The coupling reaction was diluted in 10 mL PBS pH = 7.4 and incubated at room temperature for 20 min shaking. This solution was then loaded into a disposable column and washed with 20 mL of PBS. Protein was then eluted using PBS containing 1 M NaCl in 0.3 mL fractions. Protein containing fractions were dialyzed as described above to remove the excess salt.

For PE35-MU2, we used gel filtration chromatography on a Fast Protein Liquid Chromatography apparatus. Briefly, the conjugation reaction was spun at 18,000 × *g* for 10 min and loaded into an AKTAprime Plus FPLC System (GE 11001313) using a washed HiLoad Superdex 200 PG (GE 28989335) column. One mL fractions were collected and monitored using A280 spec. Fractions containing protein were then run on SDS PAGE. All fractions containing primarily the band specific for PE35 were pooled and concentrated to less than 1 mL. This method was also used to purify Fluorescein-labeled PE35-MU2.

To assess protein-MU2 conjugation, we used either the thiol-reactive chromogenic reagent DNTB (Ellman’s reagent), the fluorescent substrate ABDF, or a non-reducing SDS PAGE gel based assay. For the DNTB assay, thiol concentrations must be above 50 μM. BSA solutions plus or minus Protein-MU2 were mixed 1:1 with a 2 mM solution of DNTB in DMSO. The reaction was then incubated at room temperature for 10 min shaking and read at 412 nm. Concentration of thiol was determined using the molar extinction coefficient of DNTB. For the gel-based assay, GZMB solutions plus or minus conjugate were purified and dialyzed as described above which allowed any free thiol to form disulfides. Reactions were run on a non-reducing SDS PAGE gel using a BioRad Mini-Protean gelcast system (BioRad 1658005) with Mini-Protean 4–15% pre-cast gels. Gels were run at 150 V for 45 min, washed once with water, stained with SimplyBlue [Thermo LC6060] protein stain, and de-stained in water. The ABDF assay was performed using a Sensolyte ABDF Assay Kit (Anaspec AS-72137)] according to manufacturer’s recommendations. The plate was read at 389/513 ex/em. Free thiol concentrations of PE35 plus or minus MU2 were determined using a GSH standard curve.

#### Fluorescein conjugation of protein-MU2 constructs

Fluorescein-labeled protein-MU2 constructs were generated by incubating proteins with NHS-fluorescein (Thermo 46410) for 1 to 2 h at room temperature in the dark. Free NHS-Fluorescein was removed using either Pierce™ Dye Removal Columns (Thermo 22858) according to manufacturer’s recommendations or via FPLC. Conjugation efficiency was determined using the ratio between A280 and A495 using the respective molar extinction coefficient for each protein, and the fluorescein conjugate.

#### PSMA enzymatic assay

Lysate from LNCaP cells (robustly PSMA positive) was generated by pelleting cells from culture, lysing them in a solution of 50 mM Tris HCl pH = 7.5, 140 mM NaCl, and 1% Triton X-100 to a concentration of 1 × 10^7^ per mL. Cells were then incubated on ice for 15 min and then spun at 18,000 × *g* for 15 min. Supernatant was then harvested and stored at − 80 °C or used immediately as a source of PSMA. The lysate was then diluted 1:10 in PBS and incubated at 37 °C for 4 h with the PSMA specific substrate N-acetyl-aspartyl-glutamate [NAAG (4 μM)] and either buffer, protein-MU2 conjugate, an unconjugated protein, or a control urea-based PSMA inhibitor, ZJ43, to validate the assay. To determine the amount of NAAG converted to N-acetyl-aspartate and free glutamate by PSMA, a fluorescent enzyme-coupled Amplex Red Glutamic Acid/Glutamate Oxidase Assay Kit (Thermo A-12221) was used according to the protocol provided by the manufacturer. This reaction was done in Costar™ 96-Well Half-Area Plates (Fischer Corning 3694) in 100 uL total volume and was incubated for 1 h at 37 °C in the dark. The plate was then read at 530/590 nm ex/em. Activity was measured in raw RFUs and inhibition curves were based off a variable 4 parameter nonlinear regression. Unconjugated and conjugated protein-MU2 PSMA inhibition was measured in terms of % untreated control activity.

#### GZMB functional assay

GZMB-specific enzymatic assay ([Biovision K168-100) was assayed according to manufacturer’s suggestions. The plate was read at 380/500 ex/em following incubation. This reaction with GZMB or GZMB-MU2 was compared to a standard curve of free AMC to calculate the amount of substrate released.

#### In vitro characterization of protein-urea conjugates

Human prostate cancer cell lines were obtained from ATCC and grown in RPMI containing 10% FBS with supplemental l-glutamine at 5 mM and were passaged weekly. PSMA[+] PIP-PC3 and PSMA[−] Flu-PC3 cells were provided by Dr. Pomper. Cells were treated with either vehicle [PBS pH = 7.4] or the protein drug conjugate of interest at doses up to 250 nM. For GZMB-MU2, doses did not exceed 100 nM due to GZMB’s ability to affect cell growth via extracellular matrix remodeling at higher concentrations^25,26^. After 5-day exposure cell growth was determined using MTT-based cell proliferation (Promega G3582) according to manufacturer’s instructions. To assess the potency of each drug, we used a non-linear, 4-parameter, normalized inhibition curve to determine the IC50. Cells were photographed using a Nikon TE200 fluorescence microscope with the Metamorph software package at indicated magnifications. Students T-tests were performed on treated cell lines compared to untreated controls to determine statistical significance.

#### Flor-protein-urea confocal uptake assay

Internalization of fluorescein-labeled GZMB-MU2 or PE35-MU2, was assessed by exposing PIP-PC3 and Flu-PC3 cells to both conjugates and evaluating fluorescein uptake via confocal microscopy to visualize compartmentalization. Confluent cells on glass microscope plates (MatTek P12G-1.5-10-F) were treated with 100 nM of the fluorescent conjugate for 2 h at 37 °C. 10 μM 2-PMPA, a competitive inhibitor of PSMA, was used to further assess PSMA specificity of the uptake. Cells were fixed and permeabilized in 0.5 mL of methanol for 30 s then treated with ProLong DAPI-containing mountant (Thermo P36962) and allowed to dry for 10 min at room temperate in the dark. A Nikon C1si True Spectral Imaging Confocal Laser Scanning Microscope System was used to visualize cells. All images were analyzed using ImageJ software. All images are at 20 × magnification unless stated otherwise.

#### In vivo* characterization of PE35-MU2*

Prostate cancer cells were suspended in 90% matrigel solution for inoculation. Either one million PIP/Flu cells or 2 million LNCaP cells were injected subcutaneously into 4–6 week old nu/nu male mice (Jackson Labs). All cell lines were purchased from ATCC with the exception of PC3-PIP and Flu cell lines, which were developed in the laboratory of Dr. Pomper. Animals were then monitored until tumors reached at least 0.1 (PIP/Flu)] or 0.3 (LNCaP) cubic centimeters in volume. We then injected 20 μg [0.8 mg/kg] of PE35-MU2 or unconjugated PE35 intratumorally on two consecutive days and then measured tumor volume and animal weight every 3 days. Animals were treated via tail vain injection (IV) with increasing dose of PE35-MU2 to assess single dose toxicity. For efficacy studies, 50 μg (2 mg/kg) IV was administered daily. Animals were sacrificed via CO2 overdose when tumors were > 1cc or if > 15% loss of body weight. Tumors were excised, weighed, and fixed in formalin. Tumor histology was then performed by the Johns Hopkins Histology Core. Serum PSA level was determined by the Johns Hopkins Clinical Chemistry Core facility via ELISA (Hybritech) from blood collected via retroorbital puncture. These studies were conducted in accordance with the ARRIVE guidelines. Students T-tests were performed on treated vs untreated animal groups to assess statistical significance.

All experimental protocols were reviewed and approved by the Biosafety Office, Health Safety and Environment Committee of the Johns Hopkins University School of Medicine. All methods were carried out in accordance with relevant guidelines and regulations. All animal procedures were performed according to protocols approved by the Institutional Animal Care and Use Committee of the Johns Hopkins University School of Medicine.

## Supplementary Information

Supplementary Figures.
