# Supplementary material for: Targeted delivery of cytotoxic proteins to prostate cancer via conjugation to small molecule urea-based PSMA inhibitors
Source: Sci Rep. 2021 Jul 21;11:14925. doi: 10.1038/s41598-021-94534-5 (PMC8295317; doi:10.1038/s41598-021-94534-5)
Supplement: Supplementary file 1 — Supplementary Figures. [file 41598_2021_94534_MOESM1_ESM.docx]

**Targeted Delivery of Cytotoxic Proteins to Prostate Cancer via Conjugation to**

**Small Molecule Urea-based PSMA Inhibitors**

^1^Rogers OC, ^2^Rosen DM , ^2^Antony L, ^2^Harper HM, ^3^Das D, ^3^Yang X, ^3^Minn I, ^3^Mease RC, ^3^Pomper MG, ^1,2^Denmeade SR^*^

The Departments of ^1^Pharmacology and Molecular Sciences, ^2^Oncology and ^3^Radiology,

The Johns Hopkins University School of Medicine, Baltimore MD

^*^Corresponding Author:

Samuel R. Denmeade

Mailing Address: Viragh Building, 201 N. Broadway, Baltimore MD, 21287

Phone: 410-955-8875; Fax: 410-614-7287

Email: [denmesa@jhmi.edu](mailto:denmesa@jhmi.edu)

Supplemental Figures

**Supplemental Figure 1.** Change in body weight following intratumoral injection of PE35-MU2 or unconjugated PE35

**
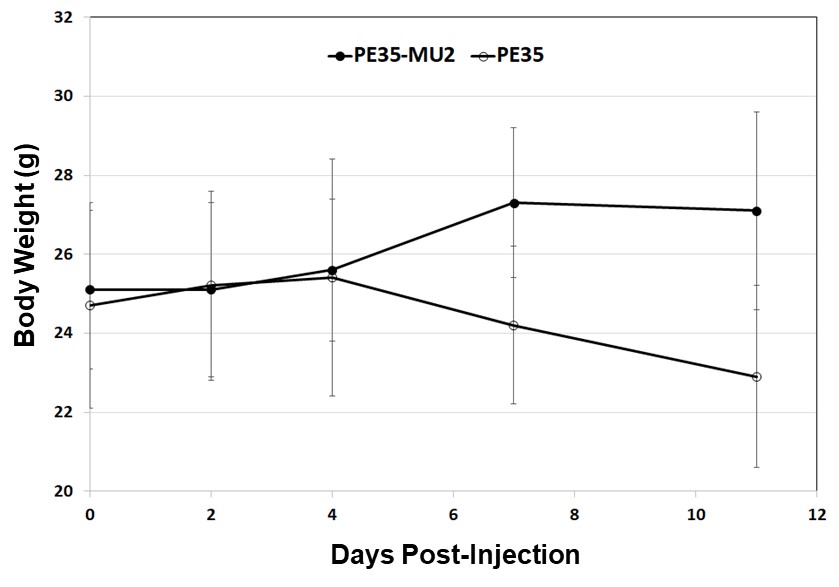
**

**Supplemental Figure 2.** (A) Growth of LNCaP xenografts following two daily injections of 50 μg (2mg/kg) PE35-MU2 (n=6) vs vehicle control (n=6) (* p=<0.05 at indicated points by students t-test).

**
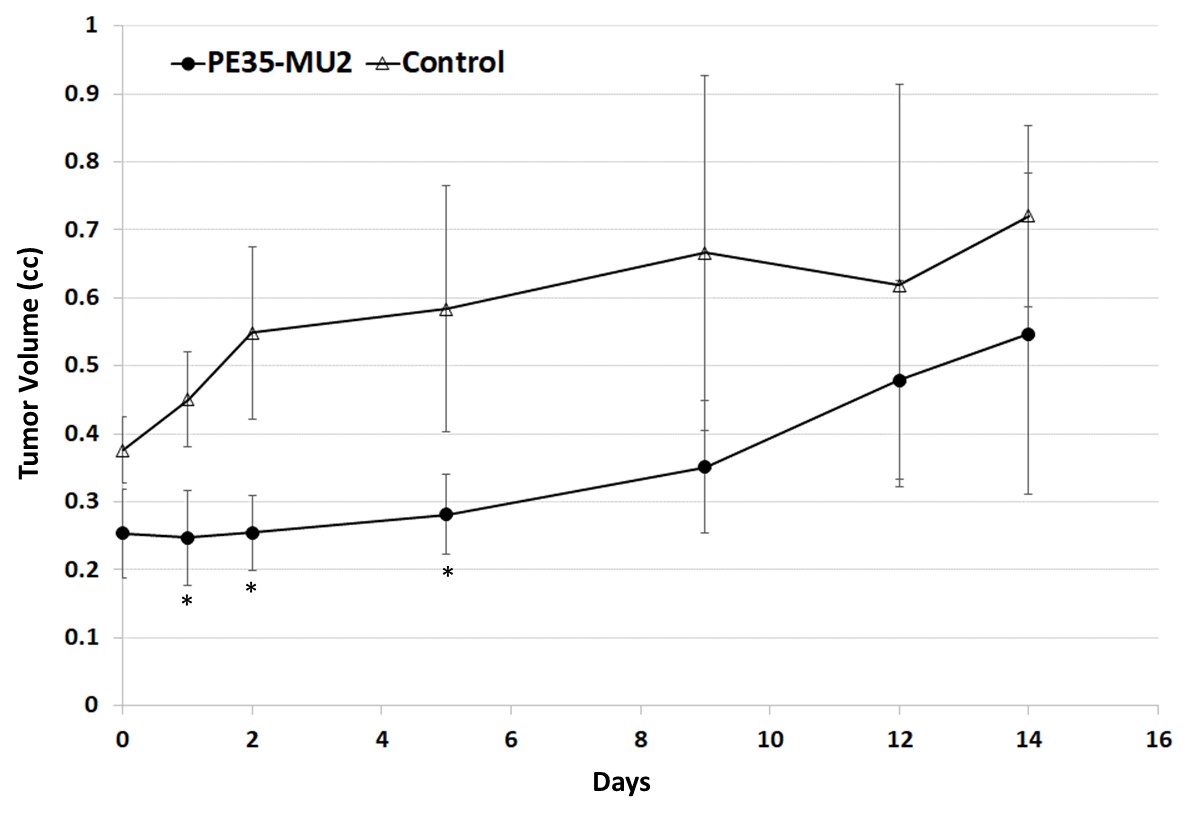
**
